# Child maltreatment and pediatric asthma: a review of the literature

**DOI:** 10.1186/s40733-016-0022-x

**Published:** 2016-04-11

**Authors:** Hannah M. C. Schreier, Edith Chen, Gregory E. Miller

**Affiliations:** 1 Department of Biobehavioral Health, The Pennsylvania State University, 219 Biobehavioral Health Building, University Park, PA 16802 USA; 2 Department of Psychology and Cells to Society (C2S): The Center on Social Disparities and Health, Institute for Policy Research, Northwestern University, Evanston, IL USA

**Keywords:** Asthma, Maltreatment, Abuse, Neglect, Children, Adolescents

## Abstract

**Background:**

Child maltreatment is a common problem with known adverse consequences, yet its contributions to the development and course of pediatric asthma are only poorly understood.

**Main:**

This review first describes possible pathways connecting child maltreatment to pediatric asthma, including aspects of the physical home environment, health behaviors and disease management, and psychological consequences of child maltreatment. We subsequently review existing studies, which generally report an association between maltreatment experiences and asthma outcomes in childhood. However, this literature is in its early stages; there are only a handful studies, most of them rely on self-reports of both child maltreatment and asthma history, and none have investigated the physiological underpinnings of this association. Taken together, however, the studies are suggestive of child maltreatment playing a role in pediatric asthma incidence and expression that should be explored further.

**Conclusion:**

Existing data are sparse and do not allow for specific conclusions. However, the data are suggestive of child maltreatment influencing asthma risk and morbidity long before the adult years. Future research should focus on understanding how child maltreatment contributes to asthma disease risk and progression in this highly vulnerable population.

## Background

Asthma remains one of the most prevalent chronic health problems facing American youth today. An estimated 14 % of children and adolescents under the age of 18 are diagnosed with asthma at some point in their lives [1]. The resulting costs are high in terms of increased rates of health care use, including physician and emergency room visits as well as hospitalizations, but also with respect to youth’s psychological well-being, academic performance, and missed school days [2–5]. Asthma has a multifactorial etiology, where genetic liabilities and environmental exposures interact in complex ways to cause reversible airway inflammation and obstruction. Though physical exposures such as viral infection, air pollution, dust mites, and cockroach antigen are known to play significant roles in asthma onset and course [6–8], mounting evidence suggests that features of the social environment do as well. In this regard, social “pollutants” such as childhood poverty, neighborhood violence, and familial stress, independently contribute to pediatric asthma outcomes, and have the potential to amplify effects of the physical environment [9–12].

Given this growing appreciation for social contributors to pediatric asthma, there is a surprising dearth of studies investigating the influence of child maltreatment history on asthma development and maintenance. Child maltreatment, including neglect as well as physical, sexual, and emotional abuse, is itself a widespread and costly problem, affecting an estimated 25 % of American youth under the age of 18 [13], with estimates of the total lifetime cost of child maltreatment estimated to be approximately $124 billion each year [14]. The extent of youth’s exposure to multiple maltreatment types is understudied but likely substantial [15, 16]. Importantly, children and adolescents who experienced child maltreatment are more likely to engage in adverse health behaviors, such as smoking, and more likely to be obese, have greater levels of chronic inflammation, and altered HPA axis profiles [17–20] - all factors known to play a role in asthma onset and course [10, 21–23]. Although long-term adverse physiological consequences of child maltreatment are increasingly being identified, very little is known about the role experiences of child maltreatment play with respect to asthma in particular. Based on what is known about the biological sequelae of child maltreatment and the importance of social contributors to asthma, however, experiences of child maltreatment are likely to play an underappreciated role in pediatric asthma.

We note that existing studies support strong links between psychological stress in general and the incidence and expression of asthma [10, 24, 25]. Nonetheless, child maltreatment represents a particularly salient adverse experience for children, and there are reasons to hypothesize its effects on asthma morbidity will be even more pronounced than other stressors, and also come in many different forms. Child maltreatment often persists over extended periods of time, involves exposure to more than one type of maltreatment, is carried out by individuals close to the victimized children, associated with both physical and psychological harm, and difficult for a child to report or take action against. Consequently, child maltreatment in particular can permeate all aspects of a child’s life and well-being. For these reasons we argue that understanding maltreatment’s asthma-related consequences will provide unique insights relevant to research and practice. In addition, a better understanding of how different types of maltreatment may or may not lead to certain asthma-related outcomes can increase our understanding of the exact situations and pathways through which adverse experiences come to negatively impact health.

This review will summarize potential pathways through which child maltreatment could influence pediatric asthma outcomes, including youth’s home environment, psychological well-being, health behaviors, and physiological pathways. Subsequently we will review the existing literature suggesting connections between child maltreatment and pediatric asthma, and discuss limitations as well as suggestions for future research.

### Potential pathways connecting child maltreatment and pediatric asthma

When considering the ways in which experiences of child maltreatment may come to impact pediatric asthma, several pathways need to be considered, including indirect effects through the physical home environment youth grow up in, disease management and individual health behaviors, and psychological consequences of maltreatment. Future research should make it a priority to disentangle the precise ways in which these other stressors confound, mediate, and moderate the association between child maltreatment and pediatric asthma. At the end of this section, the biological pathways connecting adverse social experiences to asthma-related outcomes will be reviewed briefly.

### The physical home environment

The physical home environment children grow up in contributes to pediatric asthma in important ways. Exposure to environmental tobacco smoke, indoor air pollution, and allergen exposure inside the family home are all strongly connected to pediatric asthma outcomes [7, 21, 26–29]. Convincing cross-sectional associations between common allergens, such as cockroach or mouse allergen, and asthma diagnosis as well as asthma symptoms and asthma-related hospitalizations and physician visits have been shown [7, 30]. These findings are further strengthened by reports of interventions designed to reduce allergen burdens inside the homes of youth with asthma resulting in improved clinical outcomes among these youth [31].

The physical home environment likely presents an important link between child maltreatment, especially neglect, and pediatric asthma. An estimated four out of every five cases of child maltreatment involves neglect [32]. Although there are varying definitions of child neglect, it is commonly described as the failure of caregivers to meet a child’s basic needs and provide appropriate care, food, shelter, supervision, and a safe environment [33]. Access to education and medical care are also often included. Hence, by definition, many forms of child neglect may put children at an increased risk of living in homes marked by greater chaos, less supervision, and exposure to environmental pathogens. For example, physically unsafe and dilapidated housing conditions may go hand in hand with exposure to mold, house dust, allergens, and phthalates, known contributors to asthma [7, 34–36]. Studies show that substantiated cases of child neglect frequently involve unsafe housing environments [37, 38].

Neglectful caregivers may also be less likely to engage in recommended preventive measures to successfully manage their children’s asthma and minimize their children’s symptoms, e.g., by removing carpets, frequent cleaning, and prohibiting indoor pets, or attending to their children’s health care needs regarding regular physician visits, access to current prescription medications, and daily medication adherence. Research has shown children living under inadequate housing conditions to be less likely to receive adequate physical care and to have their basic needs met [39]. Consequently, neglect in particular may negatively affect asthma management and morbidity by way of inadequate physical home environments.

### Health behaviors and asthma management

Adherence to a variety of health behaviors is an integral part of successful long-term asthma management, which frequently requires adherence to daily medication. Nonetheless, adherence rates to asthma medications are often found to be very poor [40]. Depending on children’s ages, asthma management can involve considerable involvement on the part of other family members, who help oversee daily behaviors such as proper medication adherence. In addition, certain health behaviors youth may engage in, for example smoking, can have especially detrimental consequences among youth with asthma [41, 42] who are frequently found to be more likely to smoke compared to their healthy peers [43, 44].

Again, maltreated youth may lack caregivers who can support successful disease management. They may reside in single-parent households where caregivers are overburdened, juggling multiple responsibilities, and financially stretched [45, 46], and as a result, unable to seek appropriate medical care, pay for prescribed treatments, and facilitate children’s medication adherence [38]. Parents of children with maltreatment histories are also more likely to be struggling with mental health and substance abuse problems themselves [47–49], both of which may further signal an inability on the part of parents to be actively involved in their children’s disease management process.

Youth’s own behaviors may also contribute to worse asthma outcomes. Several studies have linked experiences of child maltreatment to greater smoking rates and more persistent smoking [19, 50]. This has the potential to negatively affect both the onset of asthma as well as exaggerate existing asthma [51]. A history of child maltreatment has also been associated with increased obesity rates among children and adolescents [17]. This is of interest as obesity is increasingly being identified as a risk factor for pediatric asthma, especially nonatopic asthma, even though the underlying causes are not yet clearly understood [22, 52]. Consequently, obesity following child maltreatment may represent another pathway to increased asthma risk. So might other health practices, including drug use, sleep habits, or dietary intake, but these pathways require further investigation.

### Psychological sequelae of child maltreatment

Exposure to child maltreatment may further increase individuals’ risk for asthma through negatively impacting their psychological well-being. Several small scale studies have established preliminary connections between emotional states such as depression or anxiety and asthma-related outcomes [53, 54] and documented higher rates of behavioral problems and anxiety disorders among youth with asthma [55]. One longitudinal study, for example, linked greater rates of behavior problems to increased rates of asthma morbidity in the form of more days of wheeze and poorer functional asthma status over the course of a 9 month follow-up period [56].

The psychological consequences of child maltreatment are wide-ranging and have been well-established. Youth who experienced child maltreatment exhibit poorer emotion regulation, higher rates of internalizing and externalizing behavior problems, as well as increased rates of mood disorders, such as anxiety and depression, and finally, fewer meaningful interpersonal connections [16, 57–59]. These associations have been observed across youth exposed to different types of child abuse and neglect and may play an important role in linking child maltreatment to pediatric asthma. Depression, for example, is associated with altered endocrine and immune functions that are relevant to the course of asthma [60, 61].

Nonetheless, research suggests that not all maltreated youth experience these psychological outcomes, and that moderators such as gender of victim and timing of exposure are important to consider. Not all studies report gender differences in these associations, but in general reports of internalizing problems appear to be more common among female victims of child maltreatment, and reports of externalizing problems more common among male victims of child maltreatment [58, 59, 62]. With regard to timing of exposure, the evidence is mixed. Some studies report that maltreatment during the early childhood years (age 0–5) has the strongest influence on downstream psychological well-being [63], whereas other studies find that, depending on the exact outcome, later exposure may lead to similarly deleterious consequences [64], or that maltreatment chronicity, rather than timing of exposure, is the most important moderator of outcomes [65, 66].

### Physiological pathways

Exposure to stressors may exacerbate existing inflammatory responses mounted following exposure to environmental pathogens, e.g., traffic-related air pollution or allergens. Asthma is typically thought to be marked by a shift towards Th2-dependent processes, both an early-phase response involving IL-4 and IL-13 and a late-phase response involving IL-5. Specifically, the release of IL-4 and IL-13 sets off an inflammatory cascade involving the proliferation and differentiation of B cells, which in turn synthesize and release IgE antibodies. These bind to mast cells in the airways and, by causing them to degranulate and release histamines and leukotrienes, bring about typical asthma symptoms including smooth muscle constriction, mucus production, and edema. Similarly, IL-5 aids the production, maturation, and activation of eosinophils which contribute to airway inflammation and obstruction and are involved in longer-term asthma-related inflammatory processes.

Multiple studies have shown that stimulated production of these cytokines increases in response to experiences of stress, e.g., university examinations [67] and for children with asthma this amplified cytokine production is especially pronounced with family-related stressors, such as conflict in the parent–child relationship or chaos in the household [68]. In addition, recent studies have found that child maltreatment is associated with increased expression of IL-6, CRP [69, 70], and other nonspecific markers of low-grade inflammation, whose role in asthma is increasingly appreciated. Furthermore, a small number of studies have linked other social stressors to cytokine activity of particular relevance to asthma. For example, among youth with asthma, more chronic family and household stress is associated with increased production of the Th2 cytokines IL-5 and IL-13, and higher eosinophil counts [71]. Similarly, exposure to acute stressful life events in the context of chronic family stress has been linked to greater Th2 cytokine production, including levels of IL-4, IL-5, and IFN-gamma, among youth with asthma [72]. Although these papers do not specifically address the consequences of child maltreatment, they suggest that normative variations in family climate may shift youth’s lymphocytes towards a more aggressive Th2 phenotype, an effect that would presumably be even stronger with the more severe exposure of maltreatment.

Experiences of child maltreatment also have well-documented consequences with respect to functioning of children’s and adolescents’ hypothalamic-pituitary-adrenal (HPA) axis [20]. Importantly, HPA axis dysregulation following maltreatment has been shown to vary substantially among children, based on the type of maltreatment, age at exposure, and subsequent internalizing and externalizing symptoms, ranging from relative hypercortisolism in some to relative hypocortisolism in other cases. Glucocorticoids are important physiologic regulators of many asthma-related immune functions, and the basis of front-line asthma-control therapies for many patients. If maltreatment reduces the availability of glucocorticoids in the airways, or decreases cellular sensitivity to these molecules, there could be implications for asthma expression and/or management [73, 74]. Consistent with this possibility, one study found that among youth with asthma, experiencing both acute and chronic stress was associated with lower expression of glucocorticoid receptor mRNA in leukocytes [75], which could plausibly reduce cortisol-mediated signaling in these cells. Another study found that among youth with asthma, lower parental support was associated with higher glucocorticoid resistance, as reflected in ex vivo lymphocyte Th2 cytokine production, following co-incubation with a mitogen and hydrocortisone [76]. These patterns have not been studied in the context of maltreatment, but if present they could function as a pathway for increasing risk of asthma development or exacerbations.

## Review

### Existing studies linking child maltreatment history and pediatric asthma

We identified seven studies that report on the association of child maltreatment history and pediatric asthma, five of them focusing on cross-sectional associations and two taking advantage of longitudinal follow-up data.

Cohen et al. [77] studied a large, randomly selected sample of Puerto Rican 5–13 year olds. Child and parent-reported history of physical or sexual abuse during the past year was associated with a roughly doubled likelihood of parent-reported current asthma, as well as greater rates of asthma-related health care and medication use after controlling for a number of confounders. Independent effects of physical and sexual abuse were not reported, however, leaving unclear whether the results were driven primarily by exposure to either type of abuse or both.

Studies that have attempted to address this question have yielded contradictory findings. In a relatively large cross-sectional study of 6–7 year olds in Brazil [78], parent reports of emotional abuse were associated with symptom intensity among children with non-atopic, but not atopic, asthma. No such association was found for physical abuse after adjusting for a number of covariates. By contrast, another cross-sectional study [79] found that among 160 4–6 year olds, parent-reported physical abuse was associated with an increased risk of child asthma. Similarly, in a study of 15 year old Swedish adolescents [80], self-reported physical abuse was associated with increased self-reported asthma risk, whereas sexual abuse was not. These findings are difficult to interpret definitively. One possibility is that physical abuse predisposes youth to asthma onset, but does not play a significant role in the course of disease. Future research is needed to evaluate this further.

We also describe one cross-sectional study focused on older adolescents and young adults (16–27 year olds) from New Zealand [81]. This study is of interest in that it compared the effects of both self-reported physical and sexual abuse and official reports of maltreatment on self-reported lifetime asthma diagnosis. The authors found no association between self-reported abuse and asthma, but found that an official record of child maltreatment roughly doubled risk of asthma diagnosis. This points to the importance of considering different effects of self-reported versus official maltreatment records, possibly because official records are indicative of younger age at exposure or particularly severe cases of child maltreatment.

The strongest evidence comes from two studies using longitudinal designs to explore child maltreatment and pediatric asthma associations. Clark et al. [82] categorised 668 12–18 year olds into trauma classes based on DSM-IV PTSD Criterion A guidelines, ranging from no exposure to trauma to various physical and sexual abuse categories. Trauma class was unrelated to self-reported asthma history; no cross-sectional associations were found at baseline, or longitudinal associations at a 1 year follow-up or age 25 in this sample. These null findings may in part reflect the fact that some participants were recruited through a study of Alcohol Use Disorders. Through that project, they may have received medical, psychological, and/or addictions treatment, which may have reduced the consequences of abuse.

Only one study has directly compared the impact of different types of abuse and neglect while also taking advantage of hospital records to evaluate more objective asthma-related outcomes. Lanier et al. [83] compared large numbers of children who had at least one record of having experienced child maltreatment before age 12 to demographically similar children without abuse records and followed them for several years. After controlling for a number of individual, family, and community factors (though not asthma history or severity), reports of child maltreatment were associated with a 74–100 % greater risk of hospital treatment for asthma. In addition, multiple reports of child maltreatment were associated with more frequent asthma-related hospitalizations. Finally, the authors found no difference between the effects of history of child abuse and child neglect, suggesting that, even though the consequences of neglect are particularly understudied, they may be as important as the consequences of abuse. We note that the discrepancy between the findings of this study and the previously discussed study by Clark et al. [82] may in part be due to the focus on more severe asthma here (hospitalization for asthma based on medical records).

These few studies do not allow detailed conclusions about the extent of asthma-related consequences following experiences of child maltreatment. When taken together, they are, however, suggestive of child maltreatment playing a role in pediatric asthma. Existing results vary with respect to the differential effects of physical and sexual abuse, for example, with several studies indicating greater effects for physical abuse. This could reflect the consequences of actual physical harm incurred as a direct result of physical abuse. It may also reflect a reluctance on part of children and adolescents (or their caregivers) to report incidences of sexual abuse. Rates of sexual abuse are also lower, requiring larger samples to detect effects.

Further research is needed to clarify whether different types of maltreatment have different consequences for asthma, both in terms of its development and expression. The study by Lanier and colleagues [83] is particularly encouraging and represents an important stepping stone for future research in this area, as the authors were able to assess these associations in a large sample, using a longitudinal design and official records, and across multiple types of maltreatment. Nonetheless, the details of the links between child maltreatment and pediatric asthma have yet to be understood in all their complexity.

### Limitations of research to date

As noted above, there are several limitations to the existing studies in this area of research that complicate the interpretation of their findings. With regard to the overall association between child maltreatment and pediatric asthma, three important methodological issues arise. First, five out of the seven studies investigating the influence of child maltreatment on pediatric asthma outcomes rely solely on cross-sectional data. These designs prohibit conclusions about the direction of causality, and raise questions about whether the observed associations are reflective of maltreatment’s effects on asthma, or an alternative scenario involving reverse causality and/or third variables. For example, as described in more detail in the next sections, it is possible that asthma elicits harsh parenting, or that some of the reported associations have their origin in disadvantaged socioeconomic circumstances.

Second, a measurement issue for both child maltreatment and asthma is the heavy reliance on self- and caregiver-reports. Not only are there problems with the reliability and accuracy of self- and caregiver-reports of asthma and maltreatment history, but given that in many studies the same individual (either an older child or a caregiver) reports on both, there are additional issues of shared-method variance that may influence findings.

Third, the existing studies do not allow for the separation of effects of child maltreatment from effects of other, co-occurring stressors. Although some of the studies reviewed here made an effort to control for some co-occurring stressors e.g., parent mental health [77, 83], physical living conditions and sanitation [78], child mental health problems [79, 81–83], and domestic and community violence exposure [79], most studies did not include multiple co-occurring stressors in their analyses and no study included a specific assessment of other stressful life events that children may have been exposed to, such as parental divorce, death of a close friend or relative, experiences of bullying, and other, similar stressors.

Several additional issues relate specifically to the measurement of child maltreatment. First, few studies directly compare effects of different types of child abuse, including emotional, physical, and sexual abuse, to allow for comparison of independent effects of these different types of abuse. Second, even though the vast majority of child maltreatment involves cases of neglect, only one existing study examined the influence of neglect on pediatric asthma outcomes. As mentioned at the beginning of this paper, however, there are reasons to believe that due, for example, to influences on the physical home environment, children experiencing neglect may be at an increased risk of worse asthma outcomes. Indeed, Lanier et al. [83] found no difference between the effects of child abuse and neglect. Relatedly, many other studies [84] examine the influence of a summary score based on a count of adverse childhood events experienced. Aspects of child maltreatment are frequently included in such summary scores, but independent effects of the different types of adverse events included in the summary score are rarely reported. Consequently, it remains unclear whether differential asthma outcomes are a consequence of exposure to child maltreatment or other events included in the summary score, such as the death of a loved one or parental divorce. Third, most studies take advantage of large epidemiological data sets which only include information on whether or not children were exposed to maltreatment. More detailed information, for example on the timing and duration of exposure, is necessary to better understand the associations between child maltreatment and pediatric asthma.

Studies also vary substantially with respect to asthma measurement. The majority of studies focus simply on the presence of an asthma diagnosis. Research that considers indicators of asthma-related morbidity, health-care utilization, and functional impairment would provide a more nuanced understanding of how maltreatment relates to the child’s experience of asthma and associated costs for the medical system. Similarly, information regarding asthma management outcomes, such as the number of prescriptions filled and the type of prescriptions (e.g., rescue versus controller medication), could provide important information about the pathways connecting child maltreatment (e.g., neglect) to asthma morbidity. No studies so far have considered physiological mechanisms, such as lung functioning and relevant inflammatory processes, which could provide some understanding of how child maltreatment operates, and whether it is through physiological pathways comparable to those activated by more commonly assessed social stressors.

Finally, studies further differ with respect to the timeframe they take into account. Some studies [78, 81, 83, 82] focus on lifetime exposure to child maltreatment, others on exposure during the past 12 months [77–79]. However, given that several studies have documented adverse health effects of child maltreatment well into adulthood, inquiring only about exposure during the past year is likely to result in conservative estimates of the influence of child maltreatment as individuals grouped into ‘control/no maltreatment’ groups may have been exposed to child maltreatment earlier in life and may still be experiencing consequences. In addition, the timing of exposure should be considered carefully. It is possible that experiences of child maltreatment during particular developmental windows, such as infancy and early childhood, may have particularly strong and long-lasting effects on pediatric asthma outcomes.

### Additional important considerations

#### The role of socioeconomic status

The role socioeconomic status (SES) may play in the associations between child maltreatment and pediatric asthma also warrants consideration. Previous research has shown that children and adolescents from low SES environments are more likely to suffer from asthma and that growing up in low SES families is associated with many of the same psychological sequelae that have been shown to follow child maltreatment [9]. In fact, studies consistently list low SES as a major risk factor for experiences of both child neglect and abuse [85, 86]. Physicians, however, may also be more likely to make diagnoses of abuse when dealing with children from lower SES backgrounds, suggesting that reporting may be biased towards lower SES samples [87]. In addition, youth who experience maltreatment tend to have lower SES as adults, e.g., lower educational attainment and occupational prestige, indicating bidirectional relations between child maltreatment and achieved SES [88, 89]. Further supporting the importance of considering SES, associations between child maltreatment and later general health-related quality of life tend to shrink after careful control for SES variables [90] and there are synergistic effects of child maltreatment experiences and SES on child social and cognitive development [91]. Most studies evaluating the effect of child maltreatment on pediatric asthma have not evaluated possible interactions with SES, but this should be considered in the future. One study reported that stressful life events (including experiences of abuse) were more common among adolescents living in physical housing conditions marked by cockroaches, insecticides, dampness, and smoking, all of which have the potential to worsen asthma [84]. As children and adolescents from low SES backgrounds are at higher risk for substandard housing, general life stressors and child maltreatment, they may be particularly vulnerable to worse asthma outcomes.

#### Reverse causality

Given the largely cross-sectional nature of available data, existing studies on the influence of child maltreatment on pediatric asthma also do not allow for careful consideration of issues of reverse causality. Research has documented that children and youth whose families are under investigation for maltreatment by child welfare agencies exhibit above average rates of chronic health conditions (roughly 30–50 %, depending on the measure) [92]. Consequently, it is possible that children with chronic health conditions, such as asthma, are exposed to more abusive and neglectful behaviors from their parents, as they may require greater resources in terms of finances, emotional support, and general supervision and attention. Several studies have shown that the existence of chronic health conditions among children increases the risk of child maltreatment [93, 94]. Children’s chronic health conditions furthermore increase the risk of child maltreatment experiences across a wide range of chronic health conditions and in particular among children from lower SES families [95]. This further underscores the idea that the presence of pediatric chronic health conditions increases the strain on and puts at risk families who are already in need. These studies clearly suggest that the extent to which chronic health conditions, such as asthma, may increase the risk of child maltreatment experiences needs to be investigated more carefully.

### Recommendations for future research

Based on existing research on the impact of child maltreatment on pediatric asthma, there are several recommendations for future research. First, most data to date come from large, cross-sectional studies. However, to truly understand the connections between child maltreatment and pediatric asthma, more longitudinal data are needed.

Second, use of more in-depth study designs will be needed to understand whether particular aspects of maltreatment are influential in shaping pediatric asthma outcomes. This includes measurement of aspects of child maltreatment applicable to exposure to any subtype, such as the timing and duration of exposure. Some research suggests that the early childhood and adolescent years are times of particular vulnerability [96–98]. Other aspects worth investigating will vary as a function of the exposure studied. When focusing on child abuse, specific data such as whether or not the perpetrator was known and perceived sense of threat or sense of safety following exposure may be of interest. When focusing on child physical neglect, details on the physical home environment may provide information on how neglect comes to influence pediatric asthma. In short, a more detailed examination of potential moderators and mediators of the association between child maltreatment and asthma is needed. This may also provide insights into potential protective factors which could inform future research and prevention work.

Third, more work is needed to understand the biological pathways that might connect child maltreatment with asthma outcomes. Even though these mechanisms are understood well conceptually, more research is needed to verify them in humans. To date, no study in this area has assessed candidate physiological mediators. Hence, nothing is known on direct effects of child maltreatment on lung functioning measures among children and adolescents, or about relevant airway or allergic inflammatory processes typically associated with asthma exacerbations.

Finally, most studies to date do not report on gender effects but it is not clear that they were always examined in detail. Future research should continue to include both boys and girls and examine whether boys and girls are impacted differently by experiences of varying types of child maltreatment. At least one study has reported boys to be more likely to report general health and mental health symptoms in response to child abuse than girls [80], suggesting that there may be sex-based differences with respect to pediatric asthma as well.

## Conclusions

Although several studies link experiences of child maltreatment to adult-onset asthma and asthma morbidity among adults [99–101], very little research has investigated these associations among children and adolescents. Existing data are sparse and do not allow for specific conclusions regarding the exact associations between various types of child maltreatment and asthma-related outcomes. However, the data are suggestive of child maltreatment influencing asthma risk and morbidity long before the adult years. More research is needed to understand how child maltreatment contributes to asthma disease risk and progression in this highly vulnerable population.

## Abbreviations

CRP: c-reactive protein
HPA: Hypothalamic-pituitary-adrenal
IL: Interleukin
SES: Socioeconomic status
